# Anti-Bladder-Tumor Effect of Baicalein from *Scutellaria baicalensis* Georgi and Its Application *In Vivo*

**DOI:** 10.1155/2013/579751

**Published:** 2013-03-17

**Authors:** Jin-Yi Wu, Kun-Wei Tsai, Yi-Zhen Li, Yi-Sheng Chang, Yi-Chien Lai, Yu-Han Laio, Jiann-Der Wu, Yi-Wen Liu

**Affiliations:** ^1^Department of Microbiology, Immunology and Biopharmaceuticals, College of Life Sciences, National Chiayi University, No. 300 Syuefu Road, Chiayi 600, Taiwan; ^2^Department of Internal Medicine, Buddhist Tzuchi Dalin General Hospital, Dalin Town, Chiayi 622, Taiwan; ^3^Department of Pathology, Chiayi Christian Hospital, Chiayi 600, Taiwan

## Abstract

Some phytochemicals with the characteristics of cytotoxicity and/or antimetastasis have generated intense interest among the anticancer studies. In this study, a natural flavonoid baicalein was evaluated in bladder cancer *in vitro* and *in vivo*. Baicalein inhibits 5637 cell proliferation. It arrests cells in G1 phase at 100 **μ**M and in S phase below 75 **μ**M. The protein expression of cyclin B1 and cyclin D1 is reduced by baicalein. Baicalein-induced p-ERK plays a minor role in cyclin B1 reduction. Baicalein-inhibited p65NF-**κ**B results in reduction of cell growth. Baicalein-induced pGSK(ser9) has a little effect in increasing cyclin B1/D1 expression instead. The translation inhibitor cycloheximide blocks baicalein-reduced cyclin B1, suggesting that the reduction is caused by protein synthesis inhibition. On the other hand, neither cycloheximide nor proteasome inhibitor MG132 completely blocks baicalein-reduced cyclin D1, suggesting that baicalein reduces cyclin D1 through protein synthesis inhibition and proteasomal degradation activation. In addition, baicalein also inhibits cell invasion by inhibiting MMP-2 and MMP-9 mRNA expression and activity. In mouse orthotopic bladder tumor model, baicalein slightly reduces tumor size but with some hepatic toxicity. In summary, these results demonstrate the anti-bladder-tumor properties of the natural compound baicalein which shows a slight anti-bladder-tumor effect *in vivo*.

## 1. Introduction

Bladder cancer is the seventh most common type of cancer in worldwide man [1] and fourth in man of United States [2]. More than 90% of bladder cancers are transitional cell carcinoma (TCC), and approximately 80% of TCC belong to noninvasive papillary carcinoma that is a low-grade intraurothelial neoplasia with high recurrence. The other 20% of TCCs initiated from carcinoma in situ are at a high risk of processing to muscle invasive disease with a substantial risk for the development of distant metastasis [3, 4]. More than 10% of the low-grade papillary tumors eventually progress to high-grade muscle invasive bladder tumors. Most of the deaths from bladder cancer patients are due to invasive cancer metastasis [5], which has been a leading problem in the cancer therapy field. Multiple drugs chemotherapy has been applied for the therapy of metastatic bladder cancer; however, the adverse effect and resistance usually limit its clinical result. Therefore, some phytochemicals with the characteristics of cytotoxicity and/or antimetastasis have generated intense interest among the anticancer studies.

Baicalein, one of four major flavonoids existed in the root of *Scutellaria baicalensis* Georgi, has excellent antioxidant and anti-inflammatory activities [6, 7]. In traditional Chinese herb medicine, the root of *Scutellaria baicalensis* Georgi was usually gathered before Tomb-Sweeping Day and decocted for the purpose of “cleansing heart” and “removing toxins,” for example, cough with yellow sputum, jaundice, swelling and pain of eye, and so on. Wogonin, another one of the major flavonoids in the root of *Scutellaria baicalensis* Georgi, has been reported to reduce inflammatory cyclooxygenase-2 expression by c-Jun inhibition [8]. In addition to the anti-inflammatory effect of wogonin, baicalein has been reported to apply in cancer therapy by its cytotoxicity [9–11] and its anti-metastasis activity [12–14] recently. In human pancreatic cancer cells, 15~50 *μ*M baicalein induces apoptotic cell death through downregulation of an antiapoptotic protein Mcl-1 [11]. In human bladder cancer cells, 60~80 *μ*M baicalein retards cell growth by inhibiting CDC2 kinase activity [9]. Sixty *μ*M baicalein also induces bladder cancer cells death, but baicalein-induced p-Akt and *γ*-H2AX expression plays a protective role against cell death [10]. Moreover, 10~50 *μ*M baicalein inhibits cell migration and invasion through inhibiting MMP-2/9 activity in human hepatoma cells [12] and human breast cancer cells [13]. In human skin carcinoma, 40 *μ*M baicalein inhibits cell invasion through inhibiting an anchor protein Ezrin expression [14]. Recently, baicalein is proven to be genotoxic without producing chromosomal alterations and mutagenesis which results in the severe side effect in cancer chemotherapy [15]. According to the above data, baicalein is a candidate worth development in anticancer therapy. 

In this study, the anticancer effect of baicalein was analyzed in bladder cancer cells *in vitro* and in an orthotopic bladder tumor model *in vivo*. *In vitro*, the correlation of baicalein-induced change in Akt, ERK, p38, and p65NF-*κ*B pathways and cell viability was analyzed. *In vivo*, the antitumor effect and renal and hepatic toxicities were evaluated.

## 2. Materials and Methods

### 2.1. Cell Culture and Drug Preparation

Human bladder papillary transitional cell carcinoma 5637 cells were obtained from the Bioresource Collection and Research Center (Hsinchu, Taiwan). Mouse bladder carcinoma MB49 cells were kindly provided by Dr. Timonthy L. Ratliff (Purdue Cancer Center, *West Lafayette, IN, USA).* 5637 and MB49 cells were maintained in RPMI 1640 medium supplied with 10% fetal bovine serum (FBS), 1% penicillin, and 1% streptomycin. Cells were incubated in a CO_2_ incubator at 37°C, with 5%  CO_2_ and 95% filtered air. Baicalein was isolated from the root of *Scutellaria baicalensis* Georgi, identified [16] and dissolved in DMSO. For culture cell assay, baicalein was added in culture medium containing 0.1% DMSO. For mouse assay, baicalein was intraperitoneally injected in mice containing 10% DMSO and 90% propylene glycol (0.8 mg/100 *μ*L/mouse).

### 2.2. Reagents and Antibodies

3-(4,5-Dimethylthiazol-2-yl)-2,5-diphenyltetrazolium bromide (MTT), propidium iodide (PI), ribonuclease A (RNase A), propylene glycol, MG-132, and crystal violet were purchased from Sigma (St. Louis, MO, USA). Anti-phospho-AKT(thr308), anti-phospho-AKT(ser473), and anti-cyclin D1 antibodies were purchased from Santa Cruz (Santa Cruz, CA, USA). Anti-*α*-tubulin, anti-*β*-actin, and anti-phospho-GSK-3*β*(ser9) antibodies were purchased from GeneTex (Taichung, Taiwan). Anti-cyclin B1 was purchased from Epitomics (Burlingame, CA, USA). Anti-Bub3 was purchased from BD Biosciences (San Jose, CA, USA). Anti-p65NF-*κ*B, anti-phospho-ERK(thr202/tyr204), and anti-phospho-p38(thr180/tyr182) were purchased from Cell Signaling Technology (Danvers, MA, USA). The Millicell Hanging Cell Culture Inserts of Transwell system was purchased from Millipore (Billerica, MA, USA). Peroxidase-conjugated secondary antibodies were purchased from Jackson ImmunoResearch (West Grove, PA, USA).

### 2.3. Cell Viability Assay

Cell number was determined by colorimetric MTT assay. 5637 cells were cultured in 24-well plates at a density of 5 × 10^4^ cells/well. After 24 h, cells were incubated with various concentrations of baicalein or 0.1% DMSO for another 24~72 h. Then MTT was added into medium for 2 h, the medium was discarded, and DMSO was added to dissolve the formazan product. Each well was measured by light absorbance at 490 nm. The result was expressed as a percentage, relative to 0.1% DMSO-treated control group.

### 2.4. Cell Cycle Analysis

Around 2 × 10^6^ 5637 cells were seeded in 100 mm dishes. After 24 h incubation for attachment, baicalein or DMSO was added. After baicalein treatment for 24 h and 48 h, cells were trypsinised, centrifuged, and fixed with ice-cold 75% ethanol overnight at 4°C. After removing the ethanol, cells were stained with a DNA staining solution (containing 1 mg/mL PI and 10 mg/mL RNase A dissolved in PBS) for 30 min at room temperature. The DNA content of the stained cells was measured using a FACScan flow cytometer. The cell doublets were removed by gating the left area of FL2-W/FL2-A plot for analysis. Cell cycle data from flow cytometry was analysed using ModFit LT software.

### 2.5. Cell Migration Assay

5637 cells were seeded in 6-well plates. After cells had reached confluence, a wound was made by a 200 *μ*L plastic tip in each well. The wells were then washed twice with PBS to remove cell debris and then incubated with culture medium with DMSO (control) or baicalein. After 24 h incubation, each well was photographed by a phase contrast microscopy. The empty area was calculated by computer, and the cell mobility was calculated by (scratch area − empty area of baicalein treatment) × 100%/(scratch area − empty area of control). Measurements were performed in triplicate and presented as mean ± SE from three independent experiments.

### 2.6. Cell Invasion Assay

The invasion assay was analyzed using a Matrigel (BD Biosciences)-coated Transwell system (Millipore). The upper chamber of the transwell was coated with 25 *μ*g Matrigel. 5637 cells (1 × 10^5^) in serum-free RPMI-1640 media were seeded onto Matrigel-coated Transwell. The upper and lower chamber media were added baicalein or 0.1% DMSO. In the lower chambers, 10% FBS was added as a chemoattractant. After a 24 h incubation time, the cells that remained on the upper surface of the filter membrane were removed, and the cells on the opposite surface of the filter membrane were stained with 4% paraformaldehyde for 30 s and photographed under microscopy at 200x magnification. The number of migrated cells was counted in five randomly chosen microscope fields.

### 2.7. RT-PCR

Total RNA was isolated from cells. Reverse transcription (RT) was performed on 2 *μ*g of total RNA by 1.5 *μ*M random hexamer and RevertAid reverse transcriptase (Fermentas); then 1/20 volume of reaction mixture was used for PCR with MMP-2 specific primers (5′CTTCCAAGTCTGGAGCGATGT3′, 5′TACCGTCAAAGGGGTATCCAT3′), MMP-9 specific primers (5′AAGATGCTGCTGTTCAGCGGG3′, 5′GTCCTCTGGGCACTGCAGGAT3′), and GAPDH specific primers (5′CGGATTTGGTCGTATTGG3′, 5′AGATGGTGATGGGATTTC3′). The PCR products were analyzed by 1% agarose gel. 

### 2.8. Gelatin-Zymography Assay

The enzymatic activities of MMP-2 and MMP-9 were determined by gelatin-zymography. 3 × 10^6^ cells were seeded in 10 cm dish for 24 h and then maintained in serum-free medium with various concentrations of baicalein. The conditioned medium was collected 24 h after drug treatment, concentrated by using an Amicon Ultracel YM-10 filter. Twenty micrograms of protein obtained from the concentrated medium was mixed with nonreducing sample buffer and subjected to electrophoresis (8% SDS-PAGE copolymerize with 0.1% gelatin as substrate). The gel was washed twice (15 min/time) with 2.5% Triton X-100 and incubated at 37°C for 16~20 h in 50 mM Tris-HCl (pH 7.8), 10 mM CaCl_2_, and 0.01% NaN_3_. The gel was stained with 0.15% Coomassie brilliant blue R-250 and destained in 50% methanol and 10% acetic acid until the gelatinolytic activities were detected as clear bands against a blue background.

### 2.9. Mouse Orthotopic Bladder Tumor Model

The female C57BL/6 mice aged five to six weeks were provided by the National Laboratory Animal Center (Taipei, Taiwan) and maintained at our animal care facility for one week prior to use. The implantation of murine bladder cancer cells MB49 into C57BL/6 mice was carried out similarly as previous report [17, 18].* After MB49 inoculation (day 1), *mice were randomly assigned to two groups (10 mice/group). One group was intraperitoneally treated with vehicle (10% DMSO and 90% propylene glycol), and the other group received 0.8 mg/mouse baicalein intraperitoneally for 9 times. At the 21th day, the mice were sacrificed and the bladder volumes were measured before formalin fixation. After cutting into 4 *μ*m sections, the slides of each mouse were confirmed under a microscope in histology by hematoxylin and eosin staining. The experiment was approved by the Institutional Animal Care and Use Committee of National Chiayi University.

### 2.10. Statistical Analysis

The values shown are mean ± SEM. Data are statistically evaluated by one-way ANOVA of SigmaPlot 11.0 and shown significantly different in *P < 0.05, **P < 0.01, and ***P < 0.001.

## 3. Results

### 3.1. Cytotoxicity and Proliferation Inhibition of Baicalein in 5637 Bladder Cancer Cells

**Figure 1:**
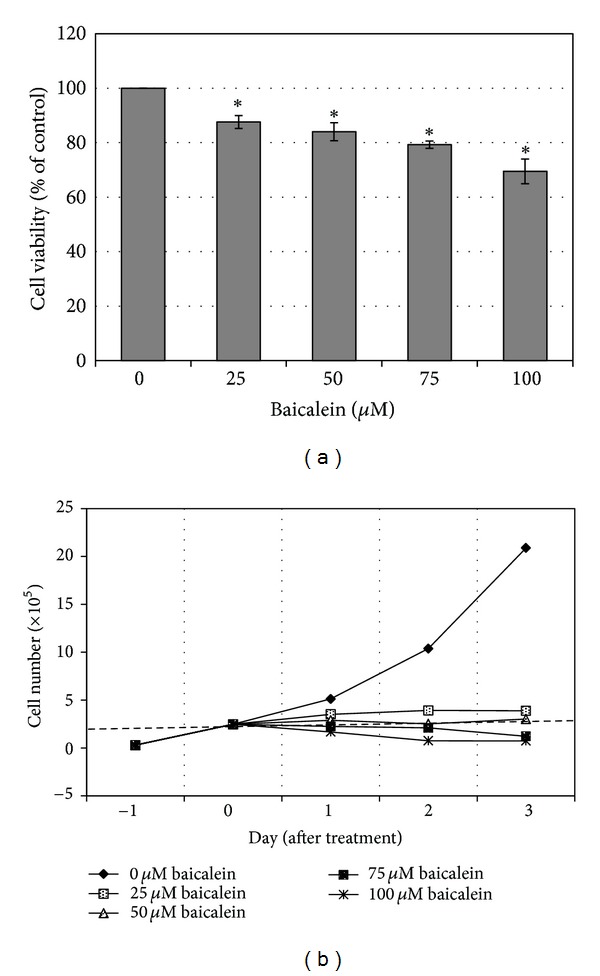
Effect of baicalein on cell growth. (a) Cytotoxicity of baicalein in 5637 bladder cancer cells. 5637 cells were initially seeded at 1 × 10^5^ cells per well in 24-well plates and then treated with various concentrations of baicalein or vehicle (0.1% DMSO) for 24 h. The cell viability was measured by MTT assay. Measurement is performed from three independent experiments (*P < 0.05 compared with vehicle). (b) Baicalein dose-dependently inhibits cell growth of 5637 cells. Cells were initially seeded at 1 × 10^5^ cells (day-1) per well in 24-well plates and then treated with various concentrations of baicalein or vehicle (0.1% DMSO) for 24~72 h. The cell number was counted by trypan blue dye exclusion assay. The dotted line indicates the cell number on day 0.

Cytotoxicity of baicalein was analyzed by MTT assay. The result shows that baicalein dose-dependently inhibits cell viability after 24 h treatment (Figure 1(a)). Below 50 *μ*M, baicalein did not induce cell death because there were no floating cells after treatment. When the concentration reached 100 *μ*M, baicalein causes 33% cell number down with dead floating cells in the culture medium. To distinguish the fact of cell death and proliferation inhibition, the direct cell count analysis was applied after baicalein treatment. The result of Figure 1(b) suggests that under 50 *μ*M, baicalein does not reduce total cell number after treatment for 72 h. Only for the concentration higher than 75 *μ*M, baicalein induces cell death dose-dependently. These data suggest that baicalein induces growth inhibition at a dose lower than 50 *μ*M and causes cell death at a dose higher than 75 *μ*M in 5637 cells.

### 3.2. Baicalein Induces Cell Cycle Arrest and Decreases Cyclin B1/D1 Expression of 5637 Bladder Cancer Cells

**Figure 2:**
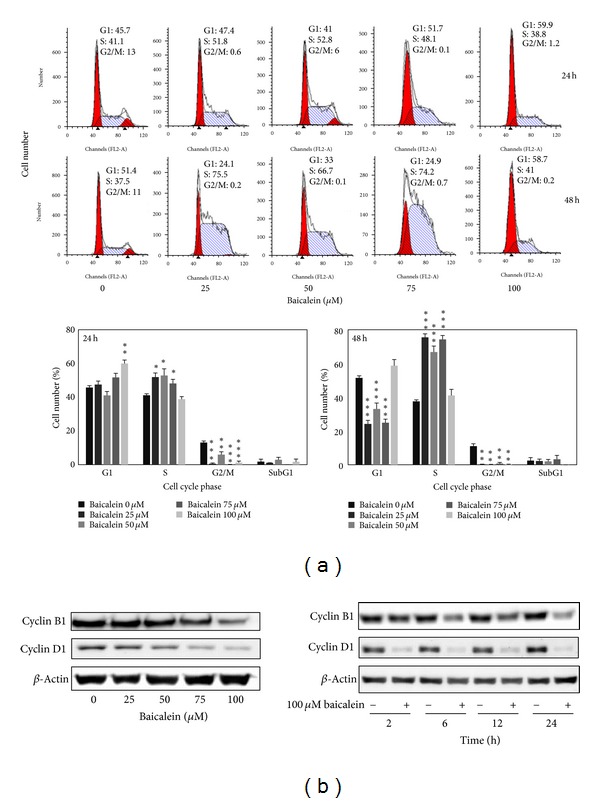
Effect of baicalein on cell cycle phase distribution in 5637 cells. (a) Baicalein induces cell cycle arrest. Cells were treated with vehicle or baicalein for 24 and 48 h, then were collected for cell cycle analysis (*P < 0.05, **P < 0.01, ***P < 0.001 compared with vehicle). (b) Effect of baicalein on cyclin B1/D1 expression. Left, cells were treated with baicalein for 24 h. Right, cells were treated with 100 *μ*M baicalein for 2, 6, 12, and 24 h.

The cell cycle distribution changed by baicalein was analyzed by flow cytometric assay. Baicalein arrests cells in S phase after 24 h treatment at the concentration under 75 *μ*M and in G1 phase at 100 *μ*M. After treatment for 48 h, 100 *μ*M baicalein continued to arrest cells in G1 without sub-G1 formation (Figure 2(a)). It suggests that baicalein, less than 50 *μ*M, caused S phase arrest without significant cytotoxicity. One hundred *μ*M baicalein arrested cells in G1 phase and induced cytotoxicity. One of G1/S transition promotion factors, cyclin D1, was dose-dependently decreased by baicalein (Figure 2(b)). It may contribute the reason to 100 *μ*M baicalein-induced G1 arrest. Baicalein also decreased cyclin B1 expression dose-dependently (Figure 2(b)). Because cyclin B1 is an essential factor for entering G2/M phase, baicalein-decreased cyclin B1 may lead to S phase arrest. Baicalein immediately decreases cyclin D1 expression after treatment for 2 h (Figure 2(b)). It suggests that 100 *μ*M baicalein effectively and quickly inhibits cell cycle progression at G1 phase.

### 3.3. Effect of Baicalein on the Regulation of Upstream Signal Factors

**Figure 3:**
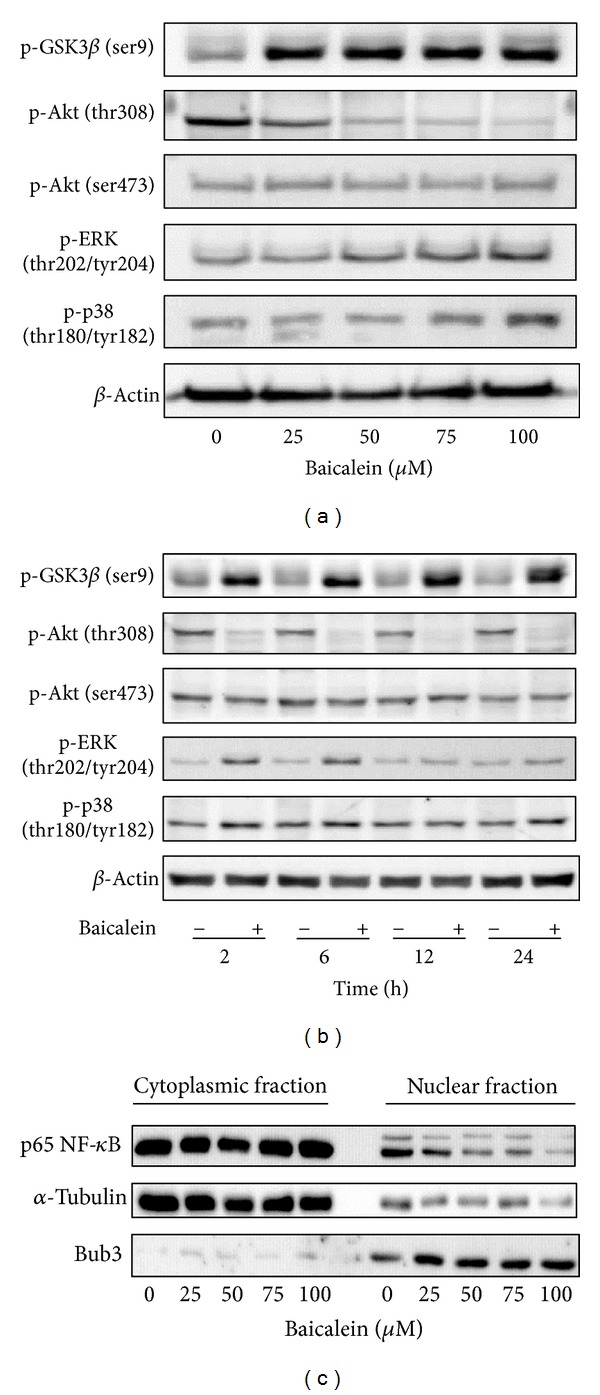
Influence of baicalein on the signal pathways. (a) Effect of baicalein on the phosphorylation of GSK3*β*, AKT, ERK, and p38. 5637 cells were treated with 0.1% DMSO or baicalein for 24 h. (b) Time-course of baicalein-changed signal protein phosphorylation. 5637 cells treated with 100 *μ*M baicalein or 0.1% DMSO for 2, 6, 12, and 24 h. The total cell lysates were extracted for western blot analysis. *β*-Actin was used as a loading control. (c) Effect of baicalein on the nuclear p65NF-*κ*B expression. 5637 cells were treated with 0.1% DMSO or baicalein for 24 h. The cytoplasmic and nuclear extracts were prepared for Western blot analysis. *α*-Tubulin and Bub3 are the loading control of cytoplasmic and nuclear fraction, respectively.

The intracellular signal factors p-GSK3*β*(ser9), p-AKT(thr308), p-AKT(ser473), p-ERK, and p-p38 were analyzed after baicalein treatment. The result of Figure 3(a) indicates that baicalein increases the phosphorylation of GSK3*β*(ser9), ERK(thr202/tyr204), and p38(thr180/tyr182). Though baicalein decreased the phosphorylation of AKT(thr308) and did not change p-AKT(ser473), the downstream GSK3*β*(ser9) was still phosphorylated at 24 h treatment. The time course of these baicalein-induced changes was also analyzed. As shown in Figure 3(b), baicalein inhibited p-AKT(thr308) phosphorylation from 2 h to 24 h; it suggests that baicalein inhibits Akt activity. GSK3*β*(ser9) was phosphorylated by baicalein from 2 h to 24 h, suggesting that baicalein also inhibits GSK3*β* activity. Both ERK and p38 pathways were early activated from 2 h to 24 h after baicalein treatment, ERK especially. The effect of baicalein on p65NF-*κ*B was also analyzed. Without extracellular stimulation, most of p65NF-*κ*B was found in cytoplasmic fraction in 5637 cells (Figure 3(c)). Baicalein dose-dependently inhibited the nuclear protein expression of p65NF-*κ*B (Figure 3(c)). In summary, baicalein inhibits AKT and GSK3*β* activities, activates ERK and p38 pathways, and inhibits p65NF-*κ*B-driven signals.

### 3.4. Effect of Various Signal Protein Inhibitors on the Baicalein-Changed Cyclin B1/D1 Expression and Cell Viability

**Figure 4:**
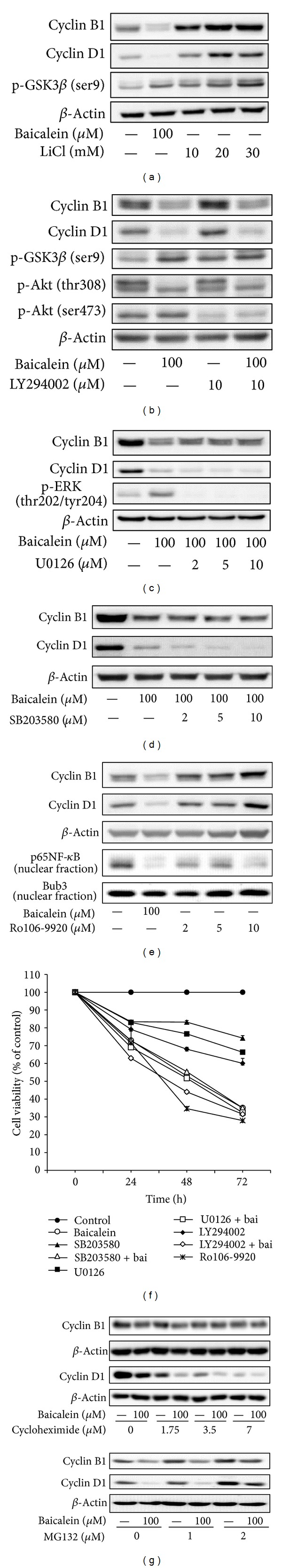
Effect of various inhibitors on baicalein-reduced cyclin B1/D1 expression. (a–e) Effect of LiCl (a), LY294002 (b), U0126 (c), SB203580 (d), and Ro106-9920 (e) on baicalein-reduced cyclin B1/D1 expression. (f) Effect of various inhibitors on baicalein-inhibited cell viability. All above inhibitors were pretreated for 1 h and baicalein treatment for 24~72 h. The concentration of each chemicals: baicalein is 100 *μ*M and others are 10 *μ*M. (g) Effect of cycloheximide or MG132 on baicalein-reduced cyclin B1/D1 expression. Cycloheximide was pretreated for 30 min and baicalein treatment for 1 h in cyclin D1 detection and baicalein treatment for 6 h in cyclin B1 detection. MG132 was pretreated for 1 h and baicalein treatment for 6 h. The extracted cell lysates or nuclear proteins were analyzed by western blot.

In order to understand the correlation between upstream signals and cyclin B1/D1 reduction, some specific inhibitors were used. Lithium chloride (LiCl) induces GSK3*β*(ser9) phosphorylation and inhibits GSK3*β* activity [19, 20]. Baicalein or LiCl increased p-GSK3*β*(ser9), but only baicalein decreased cyclin B1/D1 expression (Figure 4(a)). It suggests that baicalein-inhibited cyclin B1/D1 expression is not mediated by GSK3*β* inhibition. On the contrary, LiCl dose-dependently increased cyclin B1/D1 expression, it suggests that baicalein-inhibited GSK3*β* pathway causes cyclin B1/D1 increase instead. LY294002, the inhibitor of PI3K-Akt pathway, inhibited the phosphorylation of AKT(ser473) but increased the phosphorylation of GSK3*β*(ser9) (Figure 4(b)). However, unlike baicalein, LY294002 did not reduce cyclin B1/D1 expression (Figure 4(b)). U0126, the inhibitors of MEK-ERK, slightly reversed baicalein-decreased cyclin B1 but not cyclin D1 (Figure 4(c)). The p38 kinase inhibitor SB203580 did not reverse baicalein-decreased cyclin B1/D1 expression (Figure 4(d)). Ro106-9920, an inhibitor of p65NF-*κ*B, did not decrease cyclin B1/D1 expression (Figure 4(e)). These data indicate that baicalein-inhibited cyclin B1 is slightly mediated by ERK activation. The relationship of cell viability and baicalein-induced change in p-GSK3*β*(ser9), p-ERK, p-p38, and p65NF-*κ*B was also analyzed. Using MTT assay (Figure 4(f)), MEK-ERK inhibitor U0126 and p38 kinase inhibitor SB203580 did not affect baicalein-reduced cell viability; the PI3K inhibitor LY294002 deteriorated baicalein-reduced cell viability; the p65NF-*κ*B inhibitor Ro106-9920 reduced cell viability directly. It suggests that baicalein-induced p38, ERK, and GSK3*β*(ser9) phosphorylation does not play essential roles in cell growth inhibition. Only the baicalein-inhibited p65NF-*κ*B activity leads to reduction of cell viability. In order to find out baicalein-reduced cyclin B1/D1 caused by *de novo* protein synthesis inhibition or proteasomal degradation stimulation, the translation inhibitor cycloheximide and the proteasome inhibitor MG132 were used for this study. After cyclohexamide treatment, baicalein did not reduce cyclin B1 anymore (Figure 4(g)). But baicalein still reduced cyclin D1 expression in the presence of cycloheximide or MG132 (Figure 4(g)). It suggests both *de novo* protein synthesis inhibition and proteasomal degradation stimulation are involved in baicalein-reduced cyclin D1 expression, and cyclin B1 decrease is only caused by *de novo* protein synthesis inhibition.

### 3.5. Baicalein Blocks Migration and Invasion of 5637 Bladder Cancer Cells

**Figure 5:**
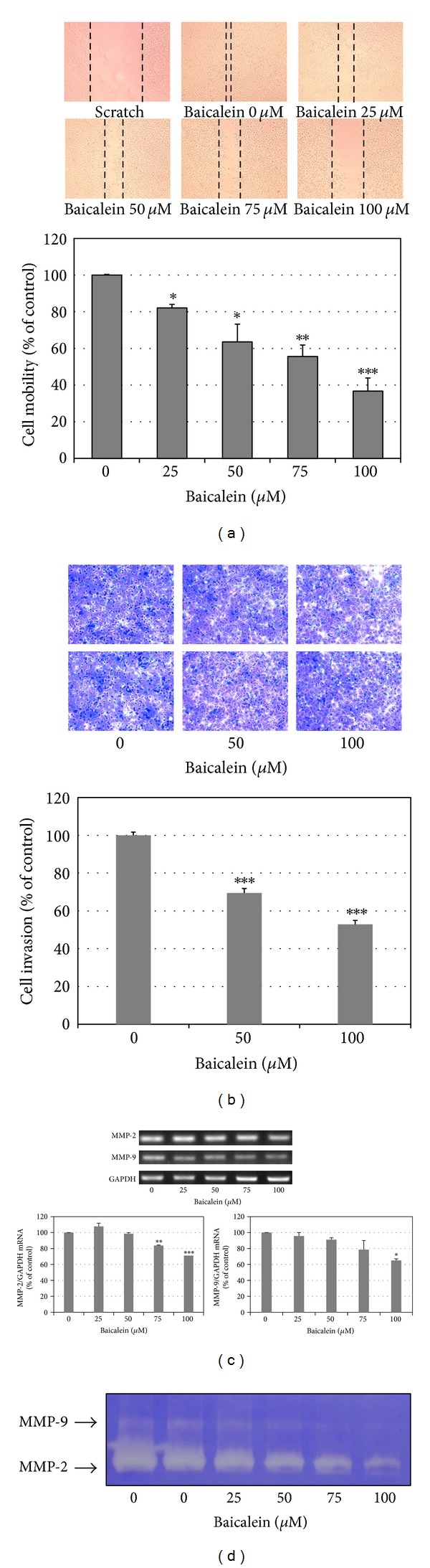
Anti-migration and anti-invasion activities of baicalein in 5637 cells. (a) Baicalein inhibits 5637 cell migration. Bottom chart is the percentage of migrated cells as control is 100%. (b) Baicalein inhibits 5637 cell invasion. Bottom chart is the percentage of invaded cells as control is 100%. (c) Effect of baicalein on the mRNA expression of MMP-2 and MMP-9. Bottom charts are the quantitative results from three independent experiments. (d) Effect of baicalein on the activities of MMP-2 and MMP-9. The conditioned medium was collected 24 h after drug treatment. Twenty micrograms of protein obtained from the concentrated medium was analyzed by gelatin-zymography assay. (*P < 0.05, **P < 0.01, ***P < 0.001 compared with vehicle).

Using scratch assay, baicalein dose-dependently inhibited cell migration (Figure 5(a)). At 100 *μ*M, baicalein shows 60% inhibition in cell migration, which is more effective than the inhibition in cell viability (33% inhibition at 100 *μ*M, Figure 1(a)). By matrigel-coated invasion assay, baicalein also shows a significant inhibition dose-dependently (Figure 5(b)). On the other hand, baicalein reduced MMP2 and MMP9 mRNA expression (Figure 5(c)) and enzymatic activity (Figure 5(d)) in 5637 cells. It suggests that the baicalein-inhibited MMP2/9 activity may contribute its anti-migration and anti-invasion activity.

### 3.6. Baicalein Slightly Inhibits Tumor Growth with Some Hepatotoxicity in a Mouse Orthotopic Bladder Tumor Model *In Vivo*

**Figure 6:**
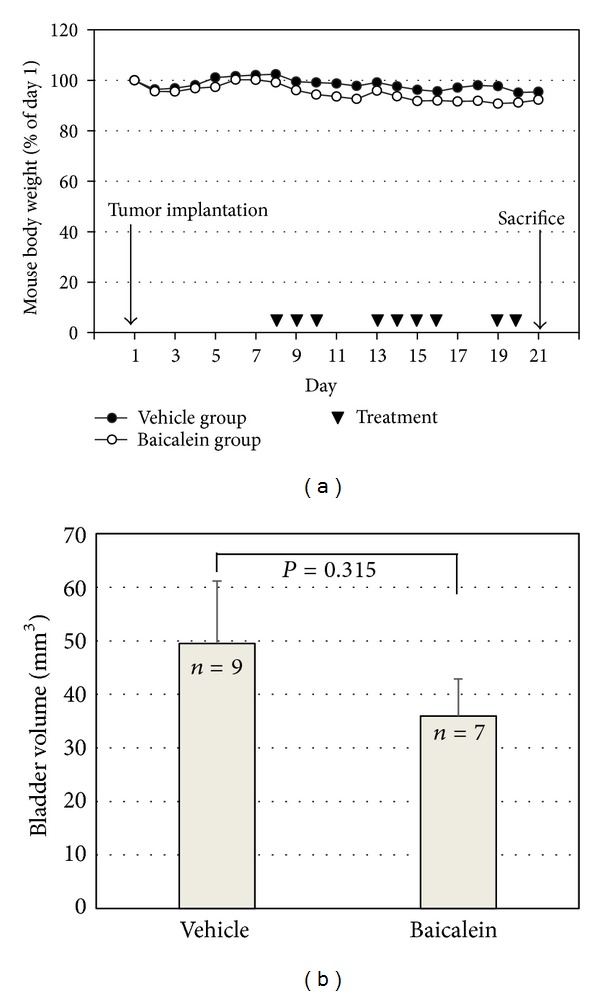
The antitumor effect of baicalein *in vivo*. (a) Mouse body weight and drug schedule in the mouse orthotopic bladder tumor model. After MB49 cell implantation, baicalein or vehicle was applied by intraperitoneal injection. Mouse body weight was recorded every day. At the 21th day, the mice were sacrificed. (b) Effect of baicalein in reducing bladder tumor size. The bladder volume of each mouse was measured. There are 9 mice survived at the 21th day in vehicle group and 7 in baicalein group.

Based on the antigrowth and antimetastasis activity of baicalein in cell assay, the *in vivo* antitumor assay was analyzed. After bladder cell implantation on day 1, baicalein treatment started on day 8. The treatment did not show toxicity in appearance and body weight (Figure 6(a)). Baicalein did not significantly reduce bladder size, but the mean bladder volume was still reduced in baicalein-treated mice (from 49.5 mm^3^ to 35.9 mm^3^ in Figure 6(b)). The blood biochemical analysis shows no significant change in serum BUN and creatinine between control and baicalein treatment groups, a little increase in GPT value but without statistical significance, and a significant increase in serum GOT (Table 1). It suggests that baicalein treatment causes some hepatic toxicity in mice.

## 4. Discussion

This study provides some new information about baicalein used in the anticancer therapy. In cell study, baicalein decreases cyclin B1 protein expression through inhibiting *de novo* protein synthesis and inhibits cyclin D1 by inhibiting protein synthesis and promoting proteasomal degradation. Baicalein-inhibited cyclin B1 is partially mediated by ERK activation. Among the signal transduction molecules AKT, GSK3*β*, ERK, p38, and p65NF-*κ*B, p65NF-*κ*B inhibition plays the most important role in baicalein-reduced cell viability. In mouse orthotopic bladder tumor model, baicalein has a little inhibition effect on orthotopic bladder tumor growth but with some hepatic toxicity.

Baicalein produces different cytotoxicity in different cell lines. For example, it causes cell cycle arrest at G1 phase in breast cancer [21] and oral squamous cell carcinoma [22], at S phase in lung nonsmall carcinoma cell [23] and at G2/M phase in BFTC905 bladder cancer cells [9]. The differences may be caused by different doses and different cells used. In 5637 bladder cancer cells, baicalein arrests cells at S phase under 75 *μ*M and at G1 phase at 100 *μ*M without apoptotic cells (Figure 2(a)). In pancreatic carcinoma PaCa cells [11], bladder cancer BFTC905 cells [9], and colorectal carcinoma HCT116 cells [10], baicalein induces cell apoptotic death at the dose between 5 to 60 *μ*M. Baicalein also has a wide range on cytotoxicity of different cell lines, the IC_50_ is under 20 *μ*M in gastric cancer cells AGS and MKN-28 [24], prostate carcinoma LNCaP [25], and JCA-1 [26], between 20 to 50 *μ*M in leukemia HL-60 [27], bladder cancer BFTC905 [9], hepatic cancer Hep G2 [28], and myeloma cell U266 [29], and more than 100 *μ*M in 5637 bladder cancer (Figure 1(a)), oral squamous carcinoma HSC-3 [22], leukemia THP-1, and osteogenic cancer cell HOS [30]. Although the detail mechanisms about the wide-range cytotoxicity are still unclear, this property may provide a specific and lower hazard anticancer effect for the higher sensitive tumors.

PI3K, the upstream signal of AKT, has been reported to be inhibited by baicalein [31]. In our study, baicalein inhibits pAKT(thr308) phosphorylation and has no influence on pAKT(ser473) (Figure 2(b)). The pAKT inhibition phenomenon also has been reported in prostate cancer cell DU145 [32] and oral squamous carcinoma HSC-3 [22]. On the other hand, the pAKT(ser473) activation has been reported in bladder cancer BFTC905 [9, 10]. Because the pAKT(thr308) phosphorylation site is the direct target site for PI3K-PDK1 [33], it will be downregulated after PI3K inhibition by baicalein (Figure 4(b)). The ser473 site of AKT is phosphorylated by rictor-mTOR [34], not PI3K; therefore, it may be the reason for the no effect of baicalein on the phosphorylation of pAKT(ser473). Even though the pAKT(thr308) is decreased by baicalein, pGSK3*β*(ser9), one AKT downstream [35], is still phosphorylated by baicalein (Figure 3(b)). Because the phosphorylation of pGSK3*β*(ser9) is achieved by numerous kinases, not only AKT [35], baicalein-induced pGSK3*β*(ser9) may be caused by the influence of other kinase(s).

NF-*κ*B, an important inflammatory transcription factor, is inhibited by baicalein in 5637 cells (Figure 3(c)). Baicalein-inhibited p65NF-*κ*B activation has also been reported in human mast cells [36], mouse macrophages [37, 38], human myeloma cells [29], and brain microglia [39, 40]. According to the important role of p65NF-*κ*B in tumor progression and metastasis [41, 42], the inhibition of baicalein on nuclear NF-*κ*B is a critical function in its anti-inflammation and anticancer application. In human hepatoma cells, baicalein shows anti-migration property with NF-*κ*B inhibition [12]. There is one report indicates that GSK3*β* inhibition results in inhibiting NF-*κ*B activity [43]; therefore, the mechanism of baicalein-inhibited p65NF-*κ*B activity may be partially mediated by baicalein-inhibited GSK3*β*. In Figure 4(f), among the 4 signal inhibitors, the cell viability decreases at most by NF-*κ*B inhibitor Ro106-9920, which indicates that NF-*κ*B is a critical factor for proliferation of 5637 cells.

Baicalein inhibits the protein expression of cyclin B1 [9, 23, 44] and cyclin D1 [22, 32, 44], has also been reported by some studies, but the mechanism is still unclear. In this study, we first suggest that baicalein decreases cyclin B1 expression through inhibiting *de novo* protein synthesis but not promoting proteasomal degradation and decreases cyclin D1 by both ways (Figure 4(g)). On the other hand, cyclin B1 reduction is partially mediated by ERK activation (Figure 4(c)). Luteolin, a natural flavonoid with structure similar to baicalein, decreases cyclin D1 expression by increasing proteasomal degradation [45]. Though the structures are similar between baicalein and luteolin, the mechanisms for cyclin D1 reduction are different. Luteolin enhances proteasomal degradation via decreasing GSK3*β*(ser9) phosphorylation, but baicalein increases GSK3*β*(ser9) phosphorylation (Figures 3(a) and 3(b)). Therefore, there is (are) other pathway(s) for inducing cyclin D1 degradation by baicalein.

In addition to anti-proliferation, baicalein also inhibits cancer cell metastasis. Either in scratch assay (Figure 5(a)) or in Matrigel-coated transwell assay (Figure 5(b)), they point out the anti-migration and anti-invasion property of baicalein. In this study, we confirm this inhibition, like others [12, 13], mediated by inhibiting MMP-2/9 activities (Figure 5(d)). The correlated signal pathways need to be further investigated. In the orthotopic bladder tumor model, baicalein shows a little effect on inhibiting bladder tumor growth (Figure 6). One report indicates that baicalein significantly reduces tumor volume in a nude mice model [12]. Comparing these two animal models, we use higher dose and lower frequency of baicalein, which may result in the lower efficiency. But it still notices that baicalein induces hepatic toxicity with GOT value increase. In order to avoid hepatic toxicity, it is better to use baicalein locally, for example, by intravesical application for bladder tumor therapy. 

## 5. Conclusions

**Figure 7:**
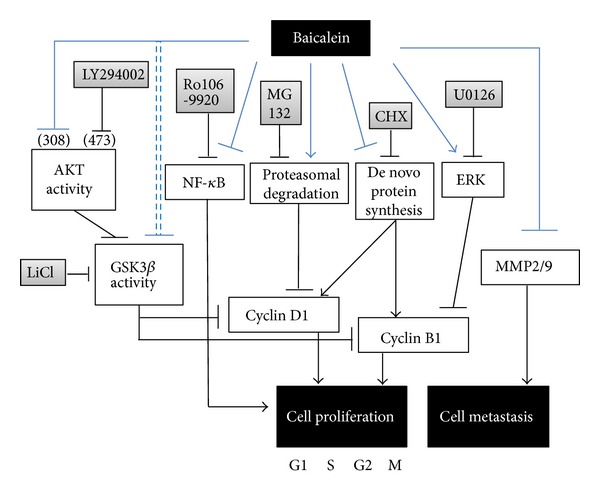
The pathway scheme of baicalein on the cell proliferation inhibition and cell metastasis inhibition in human bladder cancer cell 5637. The anti-proliferation and anti-metastasis activities of baicalein may contribute the anti-bladder-tumor effect *in vivo*.

In this study, baicalein decreases cyclin D1 protein expression through inhibiting *de novo* protein synthesis and promoting proteasomal degradation and decreases cyclin B1 by inhibiting *de novo* protein synthesis. Baicalein-inhibited cyclin B1 expression is slightly mediated by ERK activation. The mechanism of baicalein in anti-proliferation and anti-metastasis is concluded in Figure 7. Among the signal transduction molecules AKT, GSK3*β*, ERK, p38, and p65NF-*κ*B, p65NF-*κ*B inhibition plays the most important role in baicalein-reduced cell viability. In mouse orthotopic bladder tumor model, baicalein has a little effect on orthotopic bladder tumor growth inhibition but with some hepatic toxicity.

## Figures and Tables

**Table 1:** Effect of vehicle and baicalein on the plasma biochemical parameters of mice at termination of treatment. Values are mean ± SE.

	Vehicle	Baicalein
Blood urea nitrogen (BUN) (mg/dL)	22.8 ± 2.7	22.7 ± 1.8
Creatinine (mg/dL)	0.39 ± 0.01	0.39 ± 0.01
Glutamate oxaloacetate transaminase (GOT) (U/L)	66.0 ± 6.5	108.1 ± 16.6*
Glutamate pyruvate transaminase (GPT) (U/L)	27.2 ± 1.7	44.4 ± 3.6

*P < 0.05 compared with vehicle.

## Abbreviations

FBS: Fetal bovine serum
MMP-2: Matrix metalloproteinase-2
MMP-9: Matrix metalloproteinase-9
MTT: 3-(4,5-dimethylthiazol-2-yl)2,5-diphenyltetrazolium bromide
NF-*κ*B: Nuclear factor-kappaB
PI3K: Phosphatidylinositol 3-kinase.
